# DEEPGEN^TM^—A Novel Variant Calling Assay for Low Frequency Variants

**DOI:** 10.3390/genes12040507

**Published:** 2021-03-30

**Authors:** Bernd Timo Hermann, Sebastian Pfeil, Nicole Groenke, Samuel Schaible, Robert Kunze, Frédéric Ris, Monika Elisabeth Hagen, Johannes Bhakdi

**Affiliations:** 1 Department of Research & Development, Quantgene Inc. 2940 Nebraska Ave, Santa Monica, CA 90404, USA; sebastian@quantgene.com (S.P.); nicole@quantgene.com (N.G.); samuel@quantgene.com (S.S.); robert@quantgene.com (R.K.); jb@quantgene.com (J.B.); 2 Department of General, Visceral and Accident Surgery, University Hospital Heidelberg, Im Neuenheimer Feld 672, 69120 Heidelberg, Germany; 3 Department of Surgery, University Hospital Geneva, 4 Rue Gabrielle-Perret-Gentil, 1211 Geneva, Switzerland; frederic.ris@hcuge.ch (F.R.); monikahagen@aol.com (M.E.H.)

**Keywords:** variant calling, performance validation, liquid biopsy, NGS, precision medicine, early cancer detection

## Abstract

Detection of genetic variants in clinically relevant genomic hot-spot regions has become a promising application of next-generation sequencing technology in precision oncology. Effective personalized diagnostics requires the detection of variants with often very low frequencies. This can be achieved by targeted, short-read sequencing that provides high sequencing depths. However, rare genetic variants can contain crucial information for early cancer detection and subsequent treatment success, an inevitable level of background noise usually limits the accuracy of low frequency variant calling assays. To address this challenge, we developed DEEPGEN^TM^, a variant calling assay intended for the detection of low frequency variants within liquid biopsy samples. We processed reference samples with validated mutations of known frequencies (0%–0.5%) to determine DEEPGEN^TM^’s performance and minimal input requirements. Our findings confirm DEEPGEN^TM^’s effectiveness in discriminating between signal and noise down to 0.09% variant allele frequency and an LOD(90) at 0.18%. A superior sensitivity was also confirmed by orthogonal comparison to a commercially available liquid biopsy-based assay for cancer detection.

## 1. Introduction

Next generation sequencing (NGS) has become an essential technology for an array of biological and medical disciplines [1]. Cost reductions, accompanied by continuous technical advancements, have made massive parallel sequencing of the genome and transcriptome particularly interesting for advanced clinical diagnostics and precision medicine [2,3].

**Figure 1:**
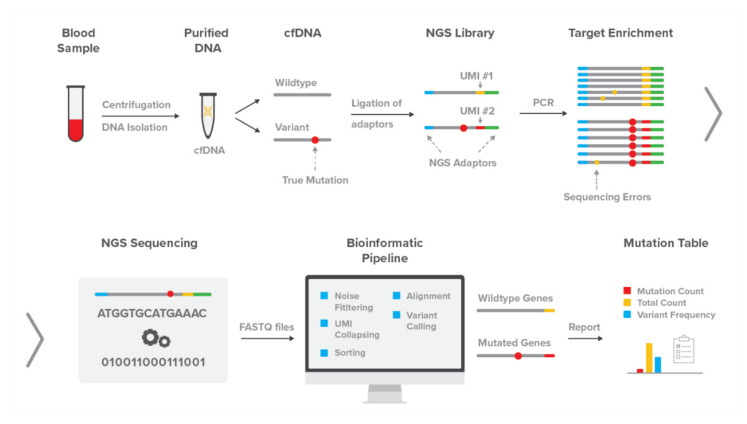
Overview of the DEEPGEN^TM^ assay workflow. Blood samples from liquid biopsies or reference materials are used to isolate cell-free DNA (cfDNA). Unique molecular identifiers (UMI) are ligated to individual cfDNA strands, followed by an enrichment of cancer-relevant sequences using a customized primer panel. The enriched NGS libraries are subsequently sequenced on a Illumina NovaSeq 6000 and analyzed with the DEEPGEN^TM^ bioinformatics pipeline. The output is a mutation table that contains the variant frequency for all targeted locations.

Detection of somatic variants provides a medically useful application of NGS technology to characterize changes at clinically relevant loci within a patient’s genome. The detection of single- and poly nucleotide variants within deoxyribonucleic acid (DNA) sequences facilitates advanced medical tasks such as diagnosing particular diseases, hereditary risk assessment, longitudinal evaluation of treatment effectiveness and gaining a deeper understanding of diseases [3,4,5]. To perform exactly these tasks with maximum efficiency, we developed DEEPGEN^TM^, a novel variant calling assay which utilizes targeted, paired-end sequencing of short reads (Figure 1). The entire assay, including the bioinformatics pipeline, were optimized to detect an extensive set of oncology-relevant variants at very low allele frequencies from liquid biopsy-derived circulating tumour DNA (ctDNA).

Especially when applied to the clinical setting, any variant calling assay needs to be reliable and demonstrate a good performance in terms of sensitivity and specificity [6]. This is exemplified in the field of precision oncology that relies on the detection of low frequency variants [7]. However, high accuracy can be hampered by several factors which need to be addressed when generating a bioinformatics pipeline for data processing and variants calling of low frequency alleles. Moreover, the laboratory processes (DNA sampling, library preparation and sequencing) are all prone to errors and can introduce systematic and stochastic noise into the data [8]. Furthermore, the efficiency of detecting a variant is also influenced by the quality of the used reference genome, the complexity of the genomic side in which the variant resides and of course by variant characteristics itself. Hence, deployed algorithmic strategies for crucial steps, such as quality-based filtering, definition of a consensus sequence or variant calling itself, can greatly impact the overall assay’s performance [9,10,11]. Lastly, the design and optimization of a bioinformatics pipeline will also be influenced by the sequencing method (whole genome, whole exome or targeted) and, thus, the targeted average coverage of a genomic position. 

Regardless of its designated application, validation of functionality and performance of a variant calling the pipeline’s performance is crucial [6,12]. Yet, evaluation of important metrics, such as sensitivity (the ratio of detected true variants) or specificity (the ability to discriminate against false positive variant calls), is often conducted in the absence of a validated ground truth. A common validation strategy attempts to determine precision of a variant calling tool by orthogonal validation against a dataset generated by another technology or by comparing multiple replicates, assuming that repeated detection, or absence of a signal can be regarded as true. However, neither approach enables the calculation of accuracy [6]. Another approach is that of synthetic datasets with artificial variants of defined frequency [13]. While this method can provide a set of true positive variants, such a reference set likely does not reflect real life data and also cannot be used to determine specificity.

This study is a technical validation of DEEPGEN^TM^. We utilized manufactured reference samples with spiked-in variants of known allele frequencies [14]. Our approach to use multiple validated variants has enabled a more comprehensive and robust testing of the DEEPGEN^TM^ assay, especially since selective omission of certain variant references provides a true negative reference and, with it, the requirement to reliably determine the accuracy of our variant calling assay.

## 2. Materials and Methods

### 2.1. The DEEPGEN^TM^ Assay

#### 2.1.1. Library Preparation and Sequencing

Reference standards (Seraseq^®^ ctDNA Mutation Mix v2, SeraCare Life Sciences Inc., Milfort, CT, USA) were purified using a Qiasymphony (Qiagen, Hilden, Germany) according to the manufacturer’s instructions. Briefly, purification procedure via QIAsymphony DSP Circulating DNA Kit (Qiagen, Hilden, Germany) comprises DNA binding, washing and elution steps, whereby the patient-like reference material (Seraseq^®^ ctDNA Mutation Mix v2) was incubated with proteinase K beforehand. Concentration of cfDNA was determined using a Tapestation 4200 (Agilent Technologies, Santa Clara, CA, USA), according to the manufacturer’s instructions. cfDNA were processed within 48 h (temporarily stored at 4 °C) and aliquots for long-term storage were kept at −80 °C.

NGS libraries were prepared from cfDNA according to the manufacturer’s instructions (Protocol based on QIAseq Targeted DNA Panel Handbook (R2; May 2017), Qiagen, Hilden, Germany). Fragmentation of the DNA was excluded from the process as the input DNA already had the optimal fragment length (200–300 nt) for targeted next generation sequencing. End-repair and Poly(A) tailing were performed, followed by QIAseqNGS adapter (QIAseq Targeted DNA Panel, Qiagen, Hilden, Germany) ligation to cfDNA molecules. Adapters comprise a sample index and a unique molecular identifier sequence (UMI), which enables merging copies of the originally captured DNA molecules during sequence analysis. After UMI attachment, target enrichment of ligated cfDNA was performed by PCR using target specific DEEPGEN^TM^ primers. The DEEPGEN^TM^ primer panel covers clinically relevant genomic targets across 272 genes, including regulatory intergenic elements. A subsequent universal PCR (using primers complementary to the adapter sequences) further amplified the cfDNA libraries and added the second sequencing adapter. 

Library concentrations were determined with KAPA Library Quantification Kits for Illumina platforms (Roche Holding AG, Basel, Switzerland) and the quality of pooled libraries were analysed using a Bioanalyzer 2100 (Agilent Technologies, Santa Clara, CA, USA). Libraries were prepared using NovaSeq Reagent Kits (Illumina, San Diego, CA, USA) and sequenced with a 300-cycle S4 kit on a NovaSeq 6000 (Illumina, San Diego, CA, USA) with a mean raw sequencing depth of ~150,000×. All sequencing steps were carried out according to the manufacturer’s instructions.

#### 2.1.2. DEEPGEN^TM^ Bioinformatics Pipeline

Sequencing data were processed with DEEPGEN^TM^’s bioinformatics pipeline. The DEEPGEN^TM^ pipeline uses FASTQ input files, created during Illumina sequencing. For each sample, the sequencing data from both paired-end reads were used to receive the most complete sequencing information possible. Only base calls with a Phred score of at least 20 were considered to have sufficient quality. In general, the quality of base calls tends to decline towards the end of a read when using sequencing by synthesis. Thus, due to the relative order of read segments, the first read is considered the main source of information. Where possible, low quality base calls in the primary read were overwritten with the respective information stored in the second read. Exception is the terminal UMI sequence, where the second read is considered the main source of information and the correction strategy is inverted.

Each resulting consensus read was then trimmed of the primer sequence, the unique molecular identifier and (if present) the constant region (CR). While the constant region represents a fixed succession of nucleotides, the primer sequence is identified by screening the first 44 bp of the read (reflecting the maximum primer length) for a sequence from DEEPGEN^TM^’s primer panel. For both, the CR and the primer sequence, DEEPGEN^TM^ is able to compensate for minor sequencing errors by using a fuzzy-match search algorithm. This method considers the editing distance (that is, the minimum number of character changes to get from string A to string B) and allows identification of a string even when up to 10% of its letters are altered (including missing or inserted nucleotides).

The specific primer sequence is used to assign the read to a defined location on the genome while the UMI provides information about the original parent DNA fragment from which the respective read originated. If the primer or UMI sequences cannot be sufficiently determined, the respective read is omitted from further processing. Consensus sequences of the original captured fragments were identified by consolidating reads based on primer and UMI information. Differing base calls introduced by sequencing errors are resolved by a majority voting system. Furthermore, a consensus sequence must be supported by at least three copies (UMI ≥ 3). Consensus sequences below this threshold are filtered and not used for variant calling.

Based on the information derived from the primer sequence, each unique consensus DNA fragment is aligned to its associated reference sequence. Reference sequences are derived from the GRCh37/hg19 [15]. Variant calling is conducted with a dynamic Smith–Waterman algorithm. This algorithm uses a semi-global alignment and tests both affine and linear gap penalty approaches, as well as different scoring schemes when determining the traceback. The alignment with the least variants was then selected. If more than twelve variants were detected, the alignment is considered “too disparate” and the DNA fragment was filtered and removed from the analysis.

Based on a whitelist with defined targets, single nucleotide variants (SNVs), multi nucleotide variants (MNPs), and short insertions/deletions (INDELS) (up to 10 base pairs) were recorded. Specific predefined longer INDELS with clinical relevance were identified with a 2nd algorithm that utilizes knowledge of the genomic position and the surrounding sequence information. Long indels are identified using a hard-coded string search, which screens for a string consisting of the actual insertion and the flanking five bp as annotated in the reference sequence. Long deletions are found by cutting the respective sequences from the reference and merging the flanking five bps into the search string. Identical genomic alterations are summarized and the count, coverage, and resulting frequency (count/coverage × 100) for each unique variant are written in a mutation table, alongside their location and mutation information. 

### 2.2. Assay Validation

#### 2.2.1. Sample Selection

Performance of the DEEPGEN^TM^ assay was determined using Seraseq^TM^ ctDNA Mutation Mix v2 reference standard (Seracare Life Sciences Inc., Milford, MA, USA). These spiked-in samples carry 40 clinically relevant mutations across 28 genes at the same specified allele frequencies. Length and composition of DNA fragments within reference standards are similar to that of real cfDNA samples. Mutations were orthogonally validated by the manufacturer using digital droplet PCR in an ISO 13485-certified and cGMP-compliant lab (Seracare Life Sciences Inc., Milford, MA, USA); 20 of the 40 Seracare mutations overlap between the Seracare and the DEEPGEN^TM^ whitelist and were used to evaluate the assay’s performance. Detailed information about the validated target variants, including exact gene locations and associated genes, can be found in the Supplementary Materials (Table S1). Reference standards encompassed both true negative variant allele frequencies (VAF of 0%; Item No 0710–0144) and true positive VAFs in differing incidences (VAFs of 0.125%, 0.25% or 0.5%; Item No 0710–0143, 0710–0142, 0710–0141). 

Library preparation and sequencing were performed in a CLIA-certified and CAP-accredited laboratory (ResearchDx, Irvine, CA, USA).

#### 2.2.2. Analytical Validation

**Figure 2:**
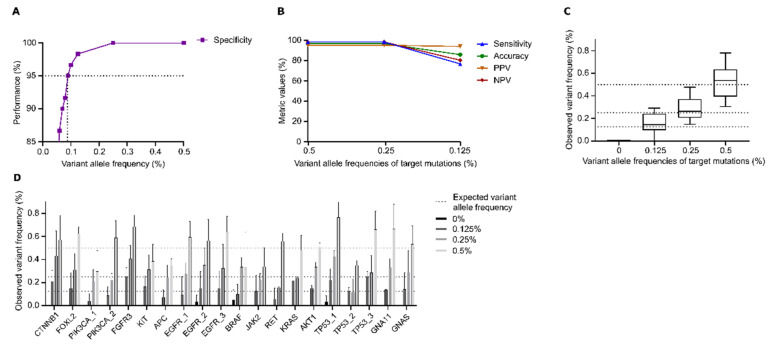
DEEPGEN^TM^ assay performance using SeraseqTM ctDNA Mutation Mix v2 reference standard with validated minor allele frequencies of 0%, 0.125%, 0.25% or 0.5% VAF as input. (**A**) The specificity of the DEEPGEN^TM^ assay is shown for a range of 0.01–0.5% VAF as threshold. The dotted line indicates the targeted quality threshold of 95%. (**B**) Sensitivity, accuracy, PPV and NPV for the verified VAFs of 0.125%, 0.25% and 0.5%. (**C**) Box plot of observed frequencies for each tested validated VAF. Whiskers represent the range of the 90 percent confidence interval. (**D**) Detected variant frequencies per validated target variant versus expected reference variant frequency. Data represent mean ± SD (*n* = 3).

Prior to DEEPGEN^TM^’s performance calculations, we heuristically determined the optimal VAF cut-off threshold, which was used to label a detected signal as true (present) or false (absent). A range of different variant frequencies was tested. The optimal VAF threshold was found at 0.09% with a very high specificity (proportion of true negatives/all negatives) of 95% (Figure 2A, Figure S1). The 0.09% VAF cut-off threshold was set as a global filter in DEEPGEN^TM^ and was thus applied to all data.

The efficiency and reliability of the DEEPGEN^TM^ assay was determined with three numerical validation experiments. We took advantage of reference standards with spiked-in variants, including true negative controls. First, reference standards for each tested VAF were sequenced and analysed in three independent replicates. True positive mutations were defined in that they were verified via DEEPGEN^TM^ and declared by the manufacturer as present, whereas false positives would refer to mutations called by DEEPGEN^TM^ in reference material at 0% VAF. True negatives were defined as variants reported as absent by the manufacturer and by DEEPGEN^TM^. The absence of expected variants was considered as false negatives. The results were used to calculate sensitivity, specificity, and accuracy, as well as a positive (PPV) and negative (NPV) predictive value (Table 1). Exact Clopper–Pearson confidence intervals for each metric were calculated using the MedCalc software (https://www.medcalc.org/calc/diagnostic_test.php (accessed on 29 March 2021), MedCalc Software, Ostend, Belgium). Results based on reference standards with 20 ng input were used to determine the limit of detection (LOD) at which the DEEPGEN^TM^ assay is still able to call 90% of all variants (LOD90). The data points with detected (1) or undetected (0) VAF were fitted with a simple logistic regression using GraphPad Prism version 8.3.1 for Windows (GraphPad Software, San Diego, CA, USA, www.graphpad.com (accessed on 29 March 2021)). The robustness of observed frequencies may differ due to varying amounts of cfDNA input. In addition, reduced input concentrations were expected to impede the detection of variants with very low allele frequencies.

In a second experiment, we tested DEEPGEN^TM^’s sensitivity with reduced input. For this, four independent replicates of reference standards with a VAF of 0.125% were sequenced, using only 5 ng input DNA, and were analysed. Based on putative differences between the standard 20 ng and the reduced 5 ng input, we further extrapolated the putative amount of input, at which at least 50% of targets could still be detected.

In the third experiment, we evaluated DEEPGEN^TM^’s intra-assay reproducibility, referring to the assay’s ability to provide robust results given the same input material. Intra-assay reproducibility was tested with six independent replicates of the Seraseq^TM^ ctDNA Mutation Mix v2 reference standard with validated target mutations at 0.5% VAF. A validated target was declared as detected when found within an acceptable range around the expected frequency (0.5 ± 0.25%). 

#### 2.2.3. Orthogonal Validation

For inter-assay variability studies, Seraseq^TM^ ctDNA Mutation Mix v2 reference standard (SeraCare Life Sciences Inc., Milford, MA, USA) was processed with the commercially available AVENIO ctDNA Surveillance assay (Roche Sequencing, Pleasanton, CA, USA) and with the DEEPGEN^TM^ assay. Both panels shared thirteen targets within the set of validated targets (details in Table S4), enabling a comparison between the assays against a known ground truth. Since the reference standards for the AVENIO assay were not sequenced in replicates, DEEPGEN^TM^’s output results were condensed for better comparison as follows: for each target at each VAF, a variant was considered as present or absent, respectively, when this finding was backed by at least two out of three replicates. For each assay, specificity, sensitivity, the PPV and NPV values were calculated for each tested VAF.

## 3. Results

### 3.1. DEEPGEN^TM^ Performance Analysis

Performance metrics of the DEEPGEN^TM^ assay (Figure 1) were calculated based on reference standards with validated allele frequencies of 0%, 0.125%, 0.25% and 0.5%. A frequency threshold of 0.09% was heuristically determined to provide the lowest, robust discrimination between true signals and sequencing artefacts, resulting in a specificity of 95% (Figure 2A).

For reference standards with a target VAF of 0.25% and 0.5%, the DEEPGEN^TM^ assay revealed sensitivity, accuracy, PPV and NPV of >95%. At 0.125% VAF, a slight drop of sensitivity, NPV and overall accuracy was noted (Figure 2B, Table 1). The bar plots show the detected variant frequency of the validated targets for VAF of 0%, 0.125%, 0.25% and 0.5% (Figure 2C). Within each tested VAF, denoted frequencies were close to the expected values. These findings are confirmed when considering the results for all 20 target variants individually: 85% of the measured VAFs lie within an accepted range of ±50% of the verified reference value (Figure 2D).

#### 3.1.1. Sensitivity of DEEPGEN^TM^

**Figure 3:**
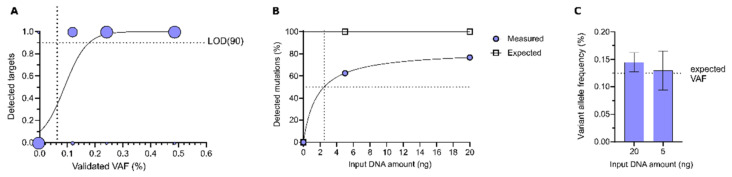
Limit of detection and minimal required input DNA material. (**A**) The number of detected (1) and absent (0) targets for each tested validated VAF across 3 replicates is shown. Circle size corresponds to the number of called variants (max = 60). Data were fitted with a logistic regression. The LOD (90) was determined at a VAF of 0.18%. (**B+C**) Comparison of 0.125% VAF reference standards with 5 ng (*n* = 4) and 20 ng (*n* = 3) input DNA. (**B**) Percentage of detected verified mutations for both tested DNA amounts. A predicted 50% detection rate was calculated for a DNA input of 2.5 ng. (**C**) Shown are the measured average variant allele frequencies of detected validated 0.125% targets. Dotted line indicates the expected VAF of 0.125%. Data represent mean ± SD (*n* = 3).

To obtain the LOD(90) for DEEPGEN^TM^, we used the number of detected targets per tested VAF and modelled the data using a logistic regression. The LOD(90) of the assay was determined at a VAF of 0.18% (Figure 3A). We further compared the variant detection rates at low frequencies based on the input DNA. With 20 ng DNA input, 77% of variants with a verified frequency of 0.125% were detected. When using 5 ng input DNA, the detection rate was diminished by ~14% to 63%. From these data we extrapolated that the predicted 50% detection threshold for a frequency of 0.125% was 2.5 ng input DNA (Figure 3B, Table S2). Between both tested input concentrations, mean frequency values of detected variants were comparably close to the expected variant frequency of 0.125%. However, when using 5 ng input DNA, individual values tended to show more variation, resulting in a slightly increased standard deviation at this DNA amount (Figure 3C). 

#### 3.1.2. Intra-Assay Reproducibility

**Figure 4:**
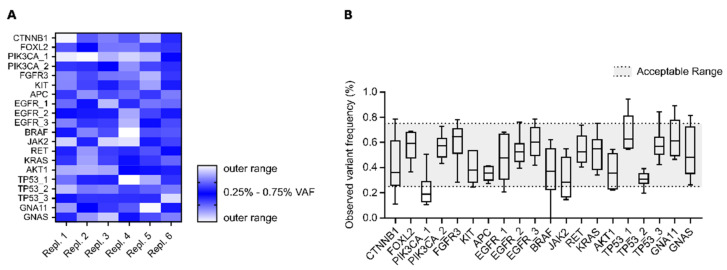
Reproducibility of DEEPGEN^TM^ assay. Results of six independent replicates of a 0.5% Seraseq^TM^ ctDNA Mutation Mix v2 reference standard were compared across 20 validated target variants. Details for each validated variant are listed in Table S3. (**A**) Heatmap representation of the six replicates with their measured variant frequencies. VAF around the expected values are shown in blue, values outside of the acceptable range (expected VAF ± 50%) in white. (**B**) Overview of all 20 validated targets (at 0.5% VAF) with their measured frequencies. The permissible range (0.25%–0. 75%) of the measured values is shown as a grey background. Box plots show the interquartile range (IQR) with whiskers representing the range of the 5–95 percent confidence interval.

To test intra-assay reproducibility, six independent replicates of the 0.5% VAF reference standard were analysed. Across these replicates, 95% of the validated variants have been detected within the target range (0.5 ± 0.25%, Figure 4A). The combined variant frequency for each reference variant across all replicates is shown (Figure 4B), which confirmed that they can be consistently measured in the acceptable frequency range of 0.5% ± 0.25%. Only one variant (PIK3CA_1) was not consistently detected or with a lower than expected VAF.

### 3.2. Orthogonal Assay Validation

**Figure 5:**
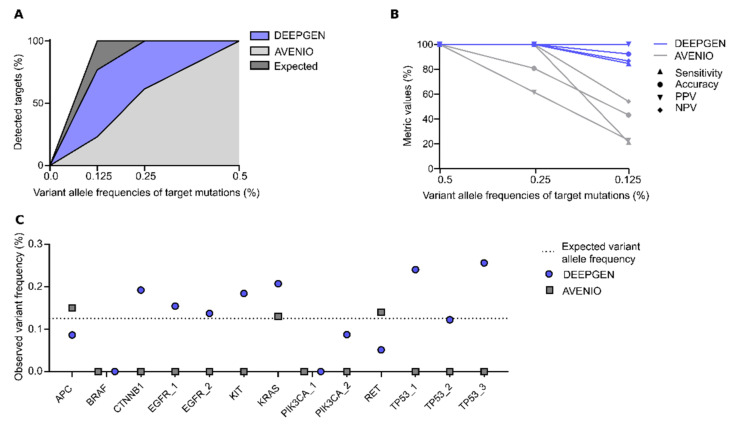
Comparison of DEEPGEN^TM^ and the AVENIO assay using 13 validated targets with known VAF. (**A**) Coloured areas represent the fraction of targets detected by DEEPGEN^TM^ (violet) and AVENIO (light grey) at 0.125%, 0.25% and 0.5% VAF. (**B**) Specificity, sensitivity, PPV, NPV and accuracy for DEEPGEN^TM^ and AVENIO assays for each tested VAF. (**C**) Overview of all 13 overlapping targets (at 0.125% VAF) with their measured variant frequencies from the AVENIO and DEEPGEN^TM^ assays.

To assess the applicability of DEEPGEN^TM^ for the detection of low frequency alleles, it was compared to the commercially available AVENIO assay [16]. Both DEEPGEN^TM^ and AVENIO pipelines did not yield any false positive variant calls and were further able to detect all validated variants at a frequency of 0.5%. At lower allele frequencies of 0.125% and 0.25%, DEEPGEN^TM^ detected 77% and 100% of targets whereas AVENIO detected 23% and 62% of targets, respectively (Figure 5A). Higher detection rates of DEEPGEN^TM^ were also reflected in overall better performance metrics (Figure 5B). In contrast to DEEPGEN^TM^’s near perfect accuracy at 0.25% VAF, Avenio achieved 80.8%. At 0.125% VAF, DEEPGENTM’s accuracy was reduced to 92.3%, while that of Avenio dropped to 43.2%. Similar trends were observed for NPV and PPV. In particular, DEEPGEN^TM^’s PPV remained at 100% while that of Avenio dropped to 23.1%, indicating the assays high variant calling reliability at low allele frequencies. Furthermore, within the measured frequencies of the individual targets, 69.2% of the values reported by DEEPGEN^TM^ and 23.1% by AVENIO were close to the expected VAF of 0.125% (±0.1%) (Figure 5C). Detailed information about targets used for orthogonal comparison, as well as assay-specific results, can be found in Table S4.

## 4. Discussion

Here, we demonstrate the DEEPGEN^TM^ assay’s high performance for the detection of variants with low allelic frequencies. The presented data suggest high variant calling accuracy and further demonstrates the pipeline’s ability to robustly discriminate between signal and noise down to a VAF of 0.09% (Figure 2A). In particular, DEEPGEN^TM^’s high sensitivity is underlined by the orthogonal validation against a commercially available assay for variant calling of low frequency alleles [17]. Furthermore, DEEPGEN^TM^’s reliability in the low VAF range is supported by the calculated LOD(90) of 0.18% as well as the projected low input required to detect 50% of low frequency targets (0.125%).Thus, this validation demonstrates that DEEPGEN^TM^ is able to reliably find genomic variants at very low allelic frequencies without negatively impacting its specificity.

Especially in the low allele frequency range, true signals and background noise become progressively harder to distinguish, which makes the comparison against a reliable baseline even more important. In this regard, the utilized reference standards not only provided a reliable baseline, but also gave confidence in the general validity of DEEPGENTM’s performance capacity, as the set of validated target variants encompassed diverse genomic loci (20 genomic locations across 15 genes). However, given the extensive nature of DEEPGEN^TM^’s primer panel and whitelist, as well as the fact that sequence complexity varies at different genomic regions, we cannot rule out the possibility that performance may be lower at certain other sites.

Nevertheless, crucial performance metrics were consistently robust across all tested VAFs. The overall high accuracy of the DEEPGEN^TM^ assay is further demonstrated in direct comparison with the AVENIO assay. Both DEEPGEN^TM^ and AVENIO were successfully able to suppress false positive variant calls, yet DEEPGEN^TM^’s detection rate was up to four times higher in the lower VAF ranges, especially at 0.125% (Figure 5). Moreover, the DEEPGEN^TM^ assay maintained an acceptable sensitivity even when challenged with a diminished amount of DNA (Figure 3). When four-fold less of the DNA input was used (20 ng to 5 ng), the number of detected variants only decreased by ~15%, demonstrating the assay’s applicability for low allele frequency variant detection from little input material.

The global cut-off frequency threshold value of 0.09% also explains the slight drop in sensitivity at 0.125% VAF and mirrors the increased difficulty to detect variants at ultra-low allelic frequencies (Figure 2B). This threshold has been heuristically determined and provided the best trade-off between sensitivity and specificity, whereas it purposely was set to be more restrictive with regards to false-positive variant calls (Figure S1). This was done since NGS-based approaches are still prone to the detection of technical artifacts that can arise from various sources and are technically difficult to control [16,18]. A recent study demonstrated that this problem is still prevalent in various popular approaches to variant calling [14]. DEEPGEN^TM^ was developed and optimized to detect a comprehensive set of cancer-relevant signals, which is why it is important to have confidence in the validity of each variant call.

While false negatives should also be avoided, the problem of missing rare variants with ultra-low frequencies has a higher relevance for other purposes, such as the survey of de novo mutations [19]. Moreover, contrary to whole-genome sequencing methods, DEEPGEN^TM^’s targeted sequence approach yields a high average coverage per targeted variant (~150,000× raw sequencing depth; mean depth after collapsing ~5000×). In NGS approaches, such as whole-genome sequencing, poor read depth can be the primary source of false-negative variants [4], which is why a high coverage strategy decreases the general chance of their occurrence.

Despite very good results, our study was confined to an analytical validation of the assay’s performance. To validate the pipeline’s use in a clinical setting, further studies with additional testing on clinical samples must be performed.

In summary, DEEPGEN^TM^ yielded excellent performance metrics for variant calling using validated reference standards, and outperformed a commercially available assay in an orthogonal comparison. Furthermore, DEEPGEN^TM^’s performance was consistent, as shown by a robust intra-assay reproducibility. Combined with a comprehensive set of clinically relevant targeted genes and variants, DEEPGEN^TM^ promises to be a valuable and precise tool for precision medicine and oncology. This may be in particular true for liquid biopsy-based diagnostics, which are usually faced with low ctDNA concentrations in patient plasma and an even lower concentration of mutant molecules [20]. Given the high accuracy for variants with very low frequencies, DEEPGEN^TM^ can be particularly useful for detecting cancer at an early stage and to monitor follow-up treatment progression using patient blood samples.

## 5. Conclusions

In conclusion, this technical validation of DEEPGEN^TM^ demonstrated the assay’s excellent performance when using industry standard reference samples containing variants with very low frequencies. Furthermore, an orthogonal comparison with an established assay highlighted DEEPGEN^TM^’s superior sensitivity for rare variant detection. The assay’s good accuracy highlights its applicability for technically challenging sampling methods, such as liquid biopsy. 

## Figures and Tables

**Table 1:** Summarized performance metrics of the DEEPGEN^TM^ assay. The sensitivity, specificity, PPV, NPV and accuracy of the variant calling was calculated for the verified VAF of 0%, 0.125%, 0.25% and 0.5%. The overall result (across validated VAFs) and the 95% confidence intervals (CI) are displayed.

Performance Metrics	Formula	VAF Specific Results		Overall Results	Overall 95% CI
Sensitivity	$Sensitivity=\left(\frac{True\;Positives}{True\;Positives+False\;Negatives}\right)\times 100$	0.5% 0.25% 0.125%	98.3% 98.3% 76.7%	91.1%	86.0%–94.8%
Specificity	$Specificity=\left(\frac{True\;Negatives}{True\;Negatives+False\;Positives}\right)\times 100$	0%	95%	95.0%	86.1%–99.0%
PPV	$PPV=\left(\frac{True\;Positives}{True\;Positives+False\;Positives}\right)\times 100$	0.5% 0.25% 0.125%	95.2% 95.2% 93.9%	98.2%	94.8%–99.6%
NPV	$NPV=\left(\frac{True\;Negatives}{True\;Negatives+False\;Negatives}\right)\times 100$	0.5% 0.25% 0.125%	98.3% 98.3% 80.3%	78.1%	66.8%–86.9%
Accuracy	$Accuracy=\left(\frac{True\;Positives+True\;Negatives}{Total\;Targets\;Examinated}\right)\times 100$	0.5% 0.25% 0.125%	96.7% 96.7% 85.8%	92.1%	87.9%–95.2%

## Data Availability

Restrictions apply to the availability of these data. Data was obtained from Quantgene Inc. and are available from the authors with the permission of Quantgene Inc.

## Supplementary Materials

The following are available online at https://www.mdpi.com/article/10.3390/genes12040507/s1, Figure S1: DEEPGEN^TM^ performance metrics at different thresholds; Table S1: Overview of the 20 validated targets; Table S2: DEEPGEN^TM^ sensitivity at different input amounts; Table S3: Intra-assay reproducibility; Table S4: Comparison of Quantgene’s DEEPGEN^TM^ with the AVENIO assay.
