# Supplementary material for: DEEPGENTM—A Novel Variant Calling Assay for Low Frequency Variants
Source: Genes (Basel). 2021 Mar 30;12(4):507. doi: 10.3390/genes12040507 (PMC8065719; doi:10.3390/genes12040507)
Supplement: Supplementary file 1 [file genes-12-00507-s001.pdf]

genes

MDPI

Supplementary Materials

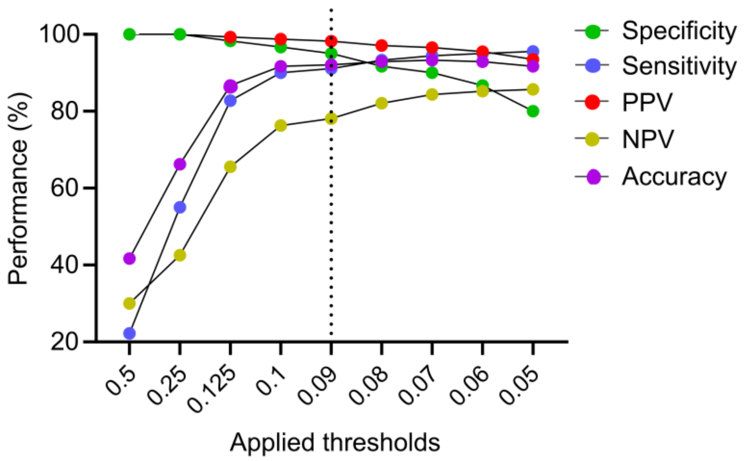

Figure S1: DEEPGENTM performance metrics at different thresholds. DEEPGENTM assay was performed with Seraseq™ ctDNA Mutation Mix v2 samples with validated minor allelic frequencies of 0.0%, 0.125%, 0.25% and 0.5% (20 ng input, n=3). Calculated are the sensitivity, specificity, the PPV and the NPA value as well as the accuracy for the DEEPGENTM assays at various VAF thresholds. A threshold of 0.09% was chosen to give the best assay performance.

Table S1: Overview of the 20 validated targets representing the overlap between the SeraCare mutation panel and the DEEPGENTM mutation panel. DEEPGENTM assay was performed with Seraseq™ ctDNA Mutation Mix v2 samples with validated minor allelic frequencies of 0.0%, 0.125%, 0.25% and 0.5% (20 ng input, n=3). Detected mutations are indicated as (1), not detected as (0). False negative and false positive values are highlighted in bold.

				0.0% VAF			0.125% VAF			0.25% VAF			0.5% VAF		
**Chr.**	**Gene**	**Location**	**Mutation**	**Rep 1**	**Rep 2**	**Rep 3**	**Rep 1**	**Rep 2**	**Rep 3**	**Rep 1**	**Rep 2**	**Rep 3**	**Rep 1**	**Rep 2**	**Rep 3**
3	CTNNB1	41266124	A/G	0	0	0	1	1	1	1	1	1	1	1	1
3	FOXL2	138665163	G/C	0	0	0	1	**0**	1	1	1	1	1	1	1
3	PIK3CA	178936091	G/A	0	0	0	1	**0**	**0**	1	1	1	1	1	1
3	PIK3CA	178952085	A/G	0	0	0	**0**	1	1	1	1	1	1	1	1
4	FGFR3	1803568	C/G	0	0	0	1	1	1	1	1	1	1	1	1
4	KIT	55599321	A/T	0	0	0	1	1	1	1	1	1	1	1	1
5	APC	112175639	C/T	0	0	0	1	1	**0**	1	1	1	1	1	1
7	EGFR	55242466	GAATTAAGAGAAGCA/-	0	0	0	1	**0**	**0**	1	1	1	1	1	1
7	EGFR	55249071	C/T	0	**1**	0	1	**0**	1	1	1	1	1	1	1
7	EGFR	55259515	T/G	0	0	0	1	1	0	1	1	1	1	1	1
7	BRAF	140453136	A/T	0	**1**	0	**0**	1	1	1	1	1	**0**	1	1

9	JAK2	5073770	G/T	0	0	0	1	1	**0**	1	1	1	1	1	1
10	RET	43617416	T/C	0	0	0	**0**	**0**	1	1	1	1	1	1	1
12	KRAS	25398284	C/T	0	0	0	1	1	1	1	1	1	1	1	1
14	AKT1	105246551	C/T	0	0	0	1	1	1	1	1	1	1	1	1
17	TP53	7577120	C/T	0	0	**1**	1	1	1	1	1	1	1	1	1
17	TP53	7577538	C/T	0	0	0	1	1	1	1	**0**	1	1	1	1
17	TP53	7578406	C/T	0	0	0	1	1	1	1	1	1	1	1	1
19	GNA11	3118942	A/T	0	0	0	1	1	1	1	1	1	1	1	1
20	GNAS	57484420	C/T	0	0	0	1	**0**	1	1	1	1	1	1	1

Table S2: DEEPGEN™ sensitivity at different input amounts. Seraseq™ ctDNA Mutation Mix v2 reference standard with validated minor allelic frequencies of 0.125% VAF and various cfDNA input (5ng and 20ng) was used for sequencing. Detected mutations are indicated as (1), not detected as (0). False negatives were highlighted in bold.

				5 ng				20 ng		
**Chr.**	**Gene**	**Location**	**Mutation**	**Rep 1**	**Rep 2**	**Rep 3**	**Rep 4**	**Rep1**	**Rep 2**	**Rep 3**
3	CTNNB1	41266124	A/G	1	**0**	**0**	1	1	1	1
3	FOXL2	138665163	G/C	**0**	1	**0**	**0**	1	**0**	1
3	PIK3CA	178936091	G/A	1	**0**	1	1	1	**0**	**0**
3	PIK3CA	178952085	A/G	1	1	1	1	**0**	1	1
4	FGFR3	1803568	C/G	1	1	1	**0**	1	1	1
4	KIT	55599321	A/T	**0**	**0**	**0**	1	1	1	1
5	APC	112175639	C/T	1	**0**	1	**0**	1	1	**0**
7	EGFR	55242466	GAATTAAGAGAAGCA/-	**0**	1	1	1	1	**0**	**0**
7	EGFR	55249071	C/T	1	1	1	1	1	**0**	1
7	EGFR	55259515	T/G	1	1	1	1	1	1	**0**
7	BRAF	140453136	A/T	**0**	1	**0**	1	**0**	1	1
9	JAK2	5073770	G/T	1	**0**	1	1	1	1	**0**
10	RET	43617416	T/C	1	**0**	1	**0**	**0**	**0**	1
12	KRAS	25398284	C/T	1	**0**	**0**	1	1	1	1
14	AKT1	105246551	C/T	1	**0**	1	**0**	1	1	1
17	TP53	7577120	C/T	1	1	1	1	1	1	1
17	TP53	7577538	C/T	**0**	1	**0**	1	1	1	1
17	TP53	7578406	C/T	1	1	1	**0**	1	1	1
19	GNA11	3118942	A/T	1	**0**	**0**	**0**	1	1	1
20	GNAS	57484420	C/T	1	**0**	**0**	1	1	**0**	1

Table S3: Intra-assay reproducibility. Six independent runs of the DEEPGEN™ assay using Seraseq™ ctDNA Mutation Mix v2 with 20 validated minor allelic frequencies of 0.5% were performed. Shown is the genome position of the 20 variants and their respective VAF [in %] of all six replicates. Shown as well is the average VAF [in %], highlighted in bold and the SD.

Chr.	Gene	Location	Mutation	Rep 1	Rep 2	Rep 3	Rep 4	Rep 5	Rep 6	Average	SD
3	CTNNB1	41266124	A/G	0.11	0.36	0.30	0.37	0.79	0.56	**0.41**	0.22
3	FOXL2	138665163	G/C	0.37	0.51	0.68	0.69	0.60	0.58	**0.57**	0.12
3	PIK3CA	178936091	G/A	0.13	0.11	0.22	0.17	0.21	0.51	**0.22**	0.14
3	PIK3CA	178952085	A/G	0.58	0.57	0.50	0.73	0.61	0.43	**0.57**	0.10
4	FGFR3	1803568	C/G	0.28	0.61	0.68	0.69	0.78	0.58	**0.61**	0.16
4	KIT	55599321	A/T	0.29	0.40	0.54	0.37	0.25	0.54	**0.40**	0.12
5	APC	112175639	C/T	0.41	0.27	0.39	0.33	0.41	0.30	**0.35**	0.06
7	EGFR	55242466	GAATTAAGAGAAGCA/-	0.33	0.52	0.21	0.43	0.68	0.67	**0.47**	0.18
7	EGFR	55249071	C/T	0.52	0.46	0.54	0.76	0.39	0.53	**0.53**	0.12
7	EGFR	55259515	T/G	0.70	0.42	0.59	0.79	0.52	0.61	**0.61**	0.12
7	BRAF	140453136	A/T	0.53	0.29	0.38	0.00	0.62	0.37	**0.37**	0.20
9	JAK2	5073770	G/T	0.17	0.55	0.17	0.15	0.46	0.40	**0.32**	0.17
10	RET	43617416	T/C	0.45	0.74	0.41	0.56	0.63	0.49	**0.55**	0.12
12	KRAS	25398284	C/T	0.58	0.75	0.40	0.52	0.59	0.34	**0.53**	0.14
14	AKT1	105246551	C/T	0.23	0.22	0.24	0.51	0.48	0.54	**0.37**	0.15
17	TP53	7577120	C/T	0.68	0.55	0.55	0.95	0.77	0.58	**0.68**	0.15
17	TP53	7577538	C/T	0.20	0.31	0.30	0.39	0.35	0.31	**0.31**	0.06
17	TP53	7578406	C/T	0.53	0.58	0.42	0.57	0.57	0.84	**0.59**	0.13
19	GNA11	3118942	A/T	0.74	0.50	0.59	0.64	0.89	0.47	**0.64**	0.15
20	GNAS	57484420	C/T	0.44	0.26	0.82	0.53	0.38	0.70	**0.52**	0.20

Table S4: Comparison of Quantgene's DEEPGEN™ with the AVENIO assay over 13 validated targets with four different VAFs (0%, 0.125%, 0.25%, 0.5%). Detected mutations are indicated as (1), not detected as (0). False negatives were highlighted in bold.

				0% VAF		0.125% VAF		0.25% VAF		0.5% VAF	
Chr.	Gene	Location	Mutation	DEEPGEN™	AVENIO	DEEPGEN™	AVENIO	DEEPGEN™	AVENIO	DEEPGEN™	AVENIO
5	APC	112175639	C/T	0	0	1	1	1	1	1	1
7	BRAF	140453136	A/T	0	0	**0**	**0**	1	1	1	1
3	CTNNB1	41266124	A/G	0	0	1	**0**	1	1	1	1
7	EGFR	55249071	C/T	0	0	1	**0**	1	1	1	1
7	EGFR	55259515	T/G	0	0	1	**0**	1	1	1	1

4	KIT	55599321	A/T	0	0	1	**0**	1	1	1	1
12	KRAS	25398284	C/T	0	0	1	1	1	1	1	1
3	PIK3CA	178936091	G/A	0	0	**0**	**0**	1	**0**	1	1
3	PIK3CA	178952085	A/G	0	0	1	**0**	1	**0**	1	1
10	RET	43617416	T/C	0	0	**0**	1	1	1	1	1
17	TP53	7577120	C/T	0	0	1	**0**	1	**0**	1	1
17	TP53	7577538	C/T	0	0	1	**0**	1	**0**	1	1
17	TP53	7578406	C/T	0	0	1	**0**	1	**0**	1	1
